# Comparison of the resonance sonorheometry based Quantra® system with rotational thromboelastometry ROTEM® sigma in cardiac surgery – a prospective observational study

**DOI:** 10.1186/s12871-021-01469-5

**Published:** 2021-10-28

**Authors:** Werner Baulig, Samira Akbas, Philipp K. Schütt, Wolfgang Keul, Marija Jovic, Pascal Berdat, Stefanie von Felten, Klaus Steigmiller, Michael Thomas Ganter, Oliver M. Theusinger

**Affiliations:** 1 Department of Anesthesiology and Intensive Care Medicine, Klinik Im Park, Seestrasse 220, CH-8027 Zurich, Switzerland; 2 Institute of Anesthesiology, University Hospital, Zurich, Switzerland; 3 Department of Cardiac Surgery, Klinik Im Park, Zurich, Switzerland; 4 Department of Biostatistics, Epidemiology, Biostatistics and Prevention Institute, University of Zurich, Zurich, Switzerland; 5 Institute of Anesthesiology, Kantonsspital Winterthur, Winterthur, Switzerland; 6 Medical Department, OSEARA health care medicine, Kloten, Switzerland

**Keywords:** Resonance Sonorheometry, Viscoelastic testing, Quantra, ROTEM sigma, cardiac surgery

## Abstract

**Background:**

Measures of the sonorheometry based Quantra® viscoelastic hemostatic analyzer (HemoSonics, LCC, Charlottesville, VA, USA) were compared with corresponding results of the ROTEM® sigma device (Instrumentation Laboratory, Bedford, MA, USA).

**Methods:**

In thirty-eight patients scheduled for elective cardiac surgery between December 2018 and October 2019, blood samples were taken after induction of anesthesia (sample 1) and after heparin neutralization (sample 2) and measured on Quantra (QPlus® Cartridge) and ROTEM sigma (ROTEM® sigma complete + hep Cartridge). Clot times and clot stiffness values were recorded. Clot stiffness values of ROTEM amplitudes (A in mm) were converted to shear modulus (G) in hectoPascal (hPa): G (hPa) = (5 x A)/(100-A). Additionally, time-to-results was recorded. Spearman rank test correlation and Bland Altman analysis were performed.

**Results:**

Clot stiffness parameters of the Quantra correlated strongly with corresponding measurements of the ROTEM with *r* = 0.93 and 0.94 for EXTEM A10 vs CS and *r* = 0.94 and 0.96 for FIBTEM A10 vs FCS for sample 1 and 2, respectively. Quantra clot time correlated strongly with ROTEM INTEM CT with *r* = 0.71 for sample 1 and *r* = 0.75 for sample 2. However, Bland Altman analysis showed no agreement in all compared assays of both methods. The median time to delivery of first and complete results was significantly shorter for Quantra (412 and 658 s) compared to ROTEM sigma (839 and 1290 s).

**Conclusions:**

The Quantra showed a strong correlation with the ROTEM sigma for determining clot times and clot stiffness and the parameters assess similar aspects of clot development. However, these parameters are not directly interchangeable and implicate that separate cut-off values need to be established for users of the Quantra device.

Word count: 278.

**Trial registration:**

The study was retrospectively registered with ClinicalTrials.gov (ID: NCT04210830) at December 20th 2019.

**Supplementary Information:**

The online version contains supplementary material available at 10.1186/s12871-021-01469-5.

## Introduction

With an incidence of up to 9.5%, major bleeding remains common in cardiac surgery and makes coagulation and transfusion management based on timely diagnostics mandatory [1]. Viscoelastic hemostatic assays (VHA) provide rapid quantitative assessments of global clotting of a whole blood sample. They are commonly used to provide a prompt diagnosis of coagulopathy allowing targeted treatment of bleeding [1].

Within a transfusion algorithm in cardiac surgery, the most commonly used VHA technologies are thromboelastography (TEG® 5000, Haemonetics Inc. Boston, MA, USA) and thromboelastometry (ROTEM® delta, Instrumentation Laboratory, Bedford, MA, USA). However, there are some clinical limitations for these techniques, such as the risk of incorrect pipetting and the use of wrong reagents (e.g. volume, concentrations, expired). Additionally, the lack of consistency between devices that assess hemostatic properties by measuring shear elastic modulus strength between an oscillating pin and a blood-filled cup, as well as the time delay between sampling and delivery of the complete results, are other limitations [2–4]. Subsequently, fully automated and cartridge-based successor versions for thromboelastography (i.e. TEG® 6 s) and thromboelastometry (i.e. ROTEM® sigma complete + hep Cartridge (ROTEM)) were developed to avoid preparation errors.

Sonic estimation of elasticity via resonance (SEER) sonorheometry is a novel ultrasound-based technology that measures viscoelastic properties of a whole blood sample [5]. Recently, the fully automated SEER based Quantra® (HemoSonics, LLC, Charlottesville, VA USA) device with the QPlus® Cartridge (Quantra) has been introduced on the medical market [6]. So far, the first comparative studies have been carried out with the manual predecessor versions of established VHA devices. In particular, promising results for comparable measures of the clot stiffness were shown for ROTEM delta and the Quantra [7–9]. So far, there have been no investigations comparing the two fully automated devices, the Quantra and the ROTEM sigma.

The primary aim of the study was to evaluate the correlation and agreement of the results between the SEER sonorheometry-based Quantra analyzer and the evolved rotational thromboelastometry-based ROTEM® sigma in cardiac surgery patients. Second, the time efficiency of both devices was compared.

## Methods

This monocentric, prospective observational study was carried out on blood samples collected from patients scheduled for elective cardiac surgery between December 15, 2018 and October 30, 2019 at the Klinik Im Park, Zurich, Switzerland. Approval for conduction of the study was provided on December 11, 2018 by the local ethics committee (Kantonale Ethikkommission, Kanton Zürich, Stampfenbachstrasse 121, 8090 Zurich, Switzerland, BASEC-Nr: 2018–01799). The manuscript has been prepared following the STROBE guidelines for reporting observational studies [10]. After written informed consent, patients scheduled for elective cardiac surgery requiring cardiopulmonary bypass were included in the study. Exclusion criteria were age less than 18 years, no German language comprehension and known congenital coagulation disorders. Blood samples were taken after induction of anesthesia (sample 1) and after the termination of cardiopulmonary bypass (CPB) and heparin neutralization (sample 2). For the study, a whole blood sample of 2.7 mL was drawn into a 3.0 mL blood collection tube containing 3.2% sodium citrate for analysis by the Quantra and ROTEM. Before CPB unfractionated heparin (Pig Heparinum sodium, Drossapharm AG, Basel, Switzerland) with an initial dosage of 300 units/kg body weight was administered directly into a central vein and its effect was monitored with the ACT technique. At our institution an ACT > 480 s is necessary to start the CPB. After termination of CPB, the remaining heparin effect was reversed with protamine (protamine hydrochloride, MEDA Pharma GmbH & Co KG, Bad Homburg, Germany), according to the initially administered heparin dose.

### Quantra® system and QPlus® cartridge methodology (Quantra)

A detailed description of the Quantra and its sonorheometry technology was recently provided by Ferrante et al. [6]. The viscoelastic characteristics of blood by Quantra are based on sonic estimation of the elasticity via resonance (SEER). A focused high-frequency ultrasound pulse is transmitted into the blood sample to generate a shear wave, causing the sample to resonate. The sample’s motion pattern is analyzed from the returning echoes, thereby, the shear modulus can be calculated (expressed in hectoPascal, hPa) [11]. The Quantra® QPlus® Cartridge is a single-use plastic multi-channel cartridge. It contains beads of lyophilized reagents in four channels that facilitate four simultaneous fully automated and independent tests. Channel 1 assesses the intrinsic kaolin activated clot time (CT, seconds), Channel 2 measures the intrinsic kaolin activated clot time in the presence of heparinase (CTH, seconds), Channel 3 measures the clot stiffness in hectoPascal (CS, hPa) after activation with thromboplastin and Channel 4 estimates the fibrinogen contribution to the clot stiffness (FCS, hPa) by adding the glycoprotein IIb/IIIa inhibitor abciximab to the thromboplastin sample. Two additional parameters are calculated: the clot time ratio (CTR) and the platelet contribution to clot stiffness (PCS, hPa) [9]. CTR is calculated as the ratio of CT and CTH, and PCS is the result of subtracting FCS from CS (Table 1).

**Table 1:** Comparison between Parameters output by the Quantra and the ROTEM sigma

Quantra	Units	Reagents	Reference Range	ROTEMsigma	Units	Reagents	Reference Range
CT	sec	kaolin	113–164 s	INTEM-CT	sec	ellagic acid	138–174 s
CTH	sec	kaolin + heparinase 1	109–150 s	HEPTEM-CT	sec	ellagic acid + heparinase 1	45–173 s
CS	hPa	thromboplastin + hexadimethrine bromide	13.0–33.2 hPa	EXTEM A10	hPa	tissue factor	3.5–8.2 hPa (41–62 mm)
FCS	hPa	Thromboplastin + hexadimethrine bromide + abciximab	1.0–3.7 hpa	FIBTEM A10	hPa	Tissue factor + cytochalasin D	0.3–1.6 hPa (5–24 mm)
PCS	hPa	Subtracting FCS from CS	11.9–29.8 hPa	EXTEM A10-FIBTEM A10	hPa	Subtracting FIBTEM A10 from EXTEM A10	3.2–6.2 hPa (36–38 mm)
CTR	Unitless	Ratio of CT over CTH	N/A		Unitless		N/A

ROTEM amplitudes (mm) were converted to shear modulus (hPa) by the formula G (hPa) = (5 x A)/(100-A), as described by Solomon et al.

*Abbreviations*: *CT* Coagulation time of the intrinsic coagulation pathway in seconds, *CTH* Clot time in the presence of heparinase in seconds, *CS* Clot stiffness in hPa, *FCS* Fibrinogen contribution to the clot stiffness in hPa, *PCS* Platelet contribution to clot stiffness in hPa, *CTR* Clot time ratio of CT and CT unitless, *INTEM-CT* Clot time in seconds (time from start the test until a clot firmness of 2 mm is detected), *HEPTEM-CT* Clot time of INTEM added with heparinase in seconds, *EXTEM A10* Clot strength at 10 min after CT of the tissue factor activated test in hPa, *FIBTEM A10* Clot strength at 10 min after CT of the tissue factor activated test added with cytochalasin in hPa, *EXTEM A10* FIBTEM A10, platelet contribution to clot stiffness in hPa, *hPa* HectoPascal

### ROTEM® sigma system and the ROTEM® sigma complete + hep cartridge (ROTEM)

The ROTEM® sigma is a fully automated thromboelastometry device using cartridges with beads containing lyophilized reagents. Method comparison of ROTEM® sigma with ROTEM® delta show similar performance [12]. Four different tests run simultaneously on the four channels. Because five ROTEM tests (INTEM, EXTEM, FIBTEM, APTEM and HEPTEM) are available, two cartridge configurations are necessary, which only differ in one channel, in which either APTEM or HEPTEM is present. As coagulation starts, the oscillation becomes constrained and the resulting impedance of the pin-rotation is detected at the pin to generate a digital output. By integrated software, the digital output is transmitted into graphical display [13]. For this investigation, the following cartridge was used: INTEM (ellagic acid activated test), EXTEM (tissue factor activated test), FIBTEM (tissue factor activated test with cytochalasin D) and HEPTEM (ellagic acid activated test with heparinase). The ROTEM sigma provides the following parameters: Clot time (CT, sec), clot formation time (CFT, sec), alpha-angle, amplitudes of clot strength at 10 to 60 min after CT (A10, A20 to A60 in mm), maximum clot strength/firmness (MCF, mm) and clot strength as a percentage of the MCF, 60 min after the CT is reached (Table 1) [12].

### Data collection

The following demographic and procedural data were documented: sex, age, height, weight, antiplatelet and anticoagulant therapy (including time of preoperative discontinuation), EuroSCORE II, type of surgery performed, surgery time, bypass time. In addition, for both devices, the following operational times were documented: the time needed from blood sampling until insertion of the Cartridge (t1); the time to first result is available (t2), the time point at which INTEM CT / CT is being quantified and displayed; and time to complete results are available (t3), the time point at which HEPTEM A10 / PCS is being quantified and displayed.

The following assays of the devices were compared: INTEM CT and Clot Time (CT), HEPTEM CT and Heparinase Clot Time (CTH), INTEM CT/HEPTEM CT and Clot Time Ratio (CTR), EXTEM A10 and Clot Stiffness (CS), FIBTEM A10 and Fibrinogen Contribution to Clot Stiffness (FCS), EXTEM A10 – FIBTEM A10 and Platelet Contribution to Clot Stiffness (PCS). Quantra clot stiffness values (CS, FCS, PCS) are expressed in hPa, while corresponding ROTEM values (EXTEM A10, FIBTEM A10, EXTEM A10-FIBTEM A10) are expressed as an amplitude in mm. The relationship between amplitude (A, mm) and shear modulus (G, Pascal) is not linear. For proper comparison and evaluation of the agreement, the ROTEM amplitudes (mm) were converted to shear modulus (hPa) by the following formula: G (hPa) = (5 x A)/(100-A), as described by Solomon et al. [14].

Additionally, postoperative blood loss (mL) estimated at 6, 12 and 24 h after arrival in the ICU, cumulative packed red blood cells (PRC), fresh frozen plasma (FFP), platelets, fibrinogen (FBG) and coagulation factor FXIII concentrates applied until discharge from the ICU were recorded.

### Statistical analysis

Sample size calculation was performed with the software PASS 11 by using ‘Non-Zero Null Tests for Simple Linear Regression’ with a significance level of 5% and a power of 80% [15].

Based on the study by Reynolds et al., comparing TEG with Quantra, it was assumed that a change in the linear regression slope by 0.05 from 0.12 to 0.17 between INTEM-CT and CT of the Quantra device would be clinically relevant [16]. Furthermore, measurements of the same patient before and after the surgery were assumed independent. For ‘INTEM-CT vs. Quantra Clot Time (CT)’, the following assumptions were made: a linear regression slope between INTEM-CT and CT of 0.12, a standard deviation of 2.25 and a Pearson correlation between CT and INTEM-CT of 0.76. Then, a sample size of 68 measurements (34 patients, 2 per patient) was estimated to be adequate to show a change in slope below 0.05.

For descriptive analyses, mean and standard deviation (SD) are reported for approximately normal continuous variables, median and interquartile range (IQR) for skewed continuous variables and frequency and percentage for categorical variables.

We used descriptive statistics to compare the times measured by tabulating them per method, once for each sample and once for the joined samples. A paired t-test was performed for the joined samples to estimate the difference in means between methods per time. Potential interactions between method and sample were investigated graphically and with linear regression including a term for method (Quantra vs. ROTEM), sample (2 vs. 1) and the method × sample interaction.

For the comparison of measurements between methods, scatterplots were used to visualize the distributions and correlations. Spearman’s rank-order correlations were calculated separately for each sample. The method of Zou and Silver et al. was used to compare correlations between samples [17]. For this part, the Holm-Bonferroni method was applied to adjust for multiple testing [18]. To assess (dis-)agreement and trends, Bland-Altman analyses were performed [19]. Spearman’s rank-order correlation coefficients were calculated to quantify the correlation of blood loss with ROTEM and Quantra measurements as well as surgery time, bypass time, and platelets of the second sample. All inferential estimates are reported together with 95% confidence intervals. Missing values were addressed with complete case analysis due to very few missing observations. The analyses were conducted with R 3.6.0 [20]. According to Schober et al. the strength of association and direction of variables (correlation) was interpreted as follows: negligible (*r* = 0.00–0.10), weak (*r* = 0.10–0.39), moderate (*r* = 0.40–0.69), strong (*r* = 0.70–0.89) and very strong (0.90–1.00) [21].

## Results

**Fig. 1:**
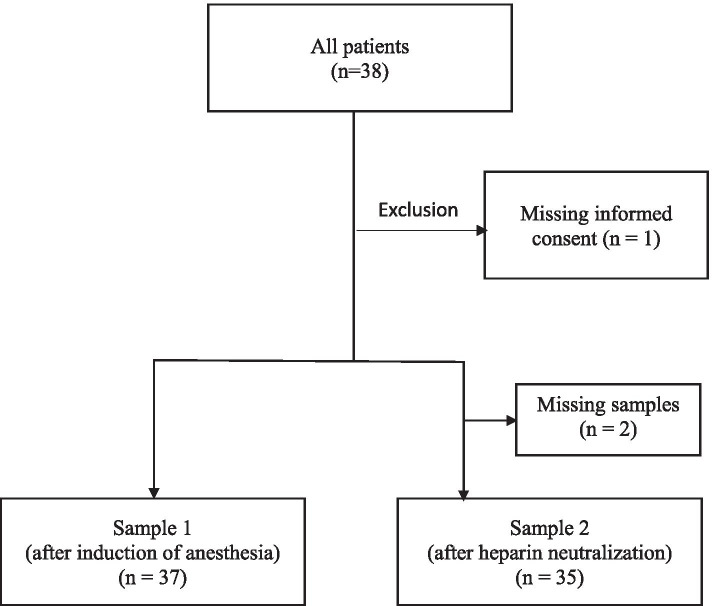
Flow chart of the study

Thirty-eight patients (thirty male and eight female) were enrolled in the study, but one male patient (two data pairs) had to be excluded because of withdrawn informed consent. In two study patients, sample 2 could not be taken because of malfunctioning vacuum tubes. Finally, a total of 72 data pairs (*n* = 37 data pairs in sample 1 and *n* = 35 data pairs in sample 2) were analyzed (Fig. 1). Patient characteristics and pre-operative treatment data are presented in Table 2. The median [IQR] EuroSCORE II was 1.7 [1.0;4.1]. In Thirty-two (86.5%) patients, the coagulation system was inhibited by low molecular weight (LMW) heparins, platelet aggregation inhibitors, direct oral anticoagulants (DOAC), Phenprocoumon or a combination of these drugs. The medication was not terminated preoperatively in patients treated with acetylsalicylic acid (*n* = 16) or LMW heparins (*n* = 3). DOAC’s and DOAC’s in combination with other anticoagulants were discontinued 2 to 4 days preoperatively in 10 patients in a median number [IQR] of 4 [2.25;4.00] days. Four patients (11.4%) experienced severe postoperative bleeding (> 1000 mL in the first 12 h). Cumulatively, seven PRC, two platelet concentrates, 16 g FBG and 2500 IE coagulation F XIII concentrate were given in these four patients. Altogether, eight patients (22.9%) received PRC, three (8.6%) FFP, four (11.4%) platelets, twelve (34.3%) FBG and four (11.4%) coagulation FXIII concentrate (Table 3).

**Table 2:** Patient baseline characteristics and pre-operative treatment

n	37	
Age; years	69.3 ± 9.5	
Height; cm	175.1 ± 8.7	
Weight; kg	83.7 ± 1 8.5	
BMI; kg.m^−2^	27.2 ± 5.0	
Sex; female	8 (21.6)	
EuroSCORE II	1.7 [1.0 to 4.1]	
**Preoperative anticoagulation/platelet inhibition**		**preoperative stop**
Acetylsalicyl acid (single drug)	16 (43.2)	0
Acetylsalicyl acid + Clopidogrel	3 (8.1)	1 (33.3)
Acetylsalicyl acid + Apixaban	1 (2.7)	1 (100)
DOAC (single drug)	5 (13.5)	5 (100)
LMWH (single drug)	3 (8.1)	0
Phenprocoumon (single drug)	2 (5.4)	1 (50)
Clopidogrel + LMWH	1 (2.7)	1 (100)
DOAC + LMWH	1 (2.7)	1 (100)
No drug given	5 (13.5)	

Data are presented as mean ± SD or median [IQR] and frequency (percentage)

*Abbreviations*: *BMI* Body mass index, *DOAC* Direct Oral Anticoagulants, *LMWH* Low Molecular Weight Heparin

**Table 3:** Procedural data

n	37
Surgery	
CABG	13 (35.1)
Aortic valve	10 (27.0)
Mitral valve	4 (10.8)
Combined intervention	10 (27.0)
Surgery time, min	400.00 [357.00 to 465.00]
Bypass time, min	122.00 [99.00 to172.00]
Blood loss after 6 h; mL	290 [120 to 505]
Blood loss after 12 h; mL	530 [315 to 690]
Blood loss after 24 h; mL	780 [600 to 1148]
Blood loss in total; mL	1010 [690 to1463]
PRC	8 (22.9)
FFP	3 (8.6)
Platelets	4 (11.4)
FBG	12 (34.3)
Factor XIII	4 (11.4)

Data for 37 patients are presented as median [IQR] or frequency (percentage)*Abbreviations*: *CABG* Coronary artery bypass graft; Aortic valve, aortic valve replacement; Mitral valve, mitral valve reconstruction or replacement; Combined intervention, combination of CABG, Valve reconstruction/replacement or aortic root surgery; *PRC* Red cell package, *FFP* Fresh frozen plasma, *FBG* Fibrinogen concentrate, *F XIII* coagulation factor XIII concentrate

**Fig. 2:**
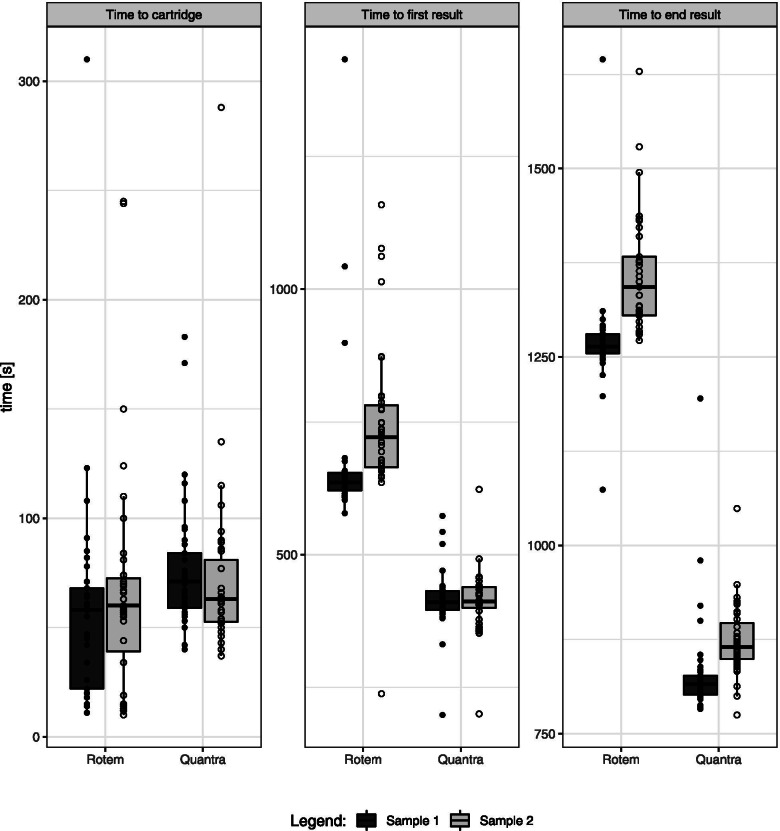
Operational times. Comparing the three different time intervals in seconds (s) of Quantra and ROTEM sigma: time to cartridge, time needed from blood sampling until insertion of the Cartridge; time to first results, time until first result was available; time to end result, time until all results were available. For both devices, boxplots in dark gray represent times for sample 1 and boxplots in light gray represent times for sample 2

The median time from blood sampling until insertion of the Cartridge (t1) did not differ between both devices (*n* = 72 data pairs). Figure 2 and Table supplement 1 show the median time [IQR] to obtain the time to first result (t2) and time to complete result (t3): it is significantly shorter for Quantra (412 [296;434] sec for t2 and 839 [810;897] sec for t3) compared to ROTEM sigma (658 [635;732] sec for t2 and 1290 [1264;1345] sec for t3).

**Fig. 3:**
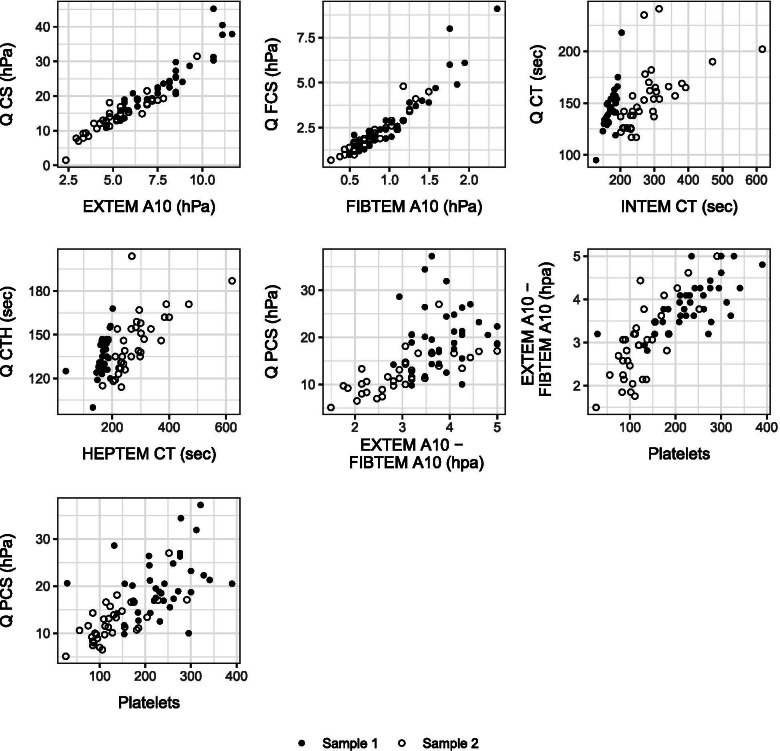
Scatterplots. Comparing Quantra and ROTEM sigma measurements as well as between Quantra/ROTEM sigma and platelet count for sample 1 and 2. Black filled circles represent measurements of sample 1, open circles represent measurements of sample 2. *Abbreviations:* CS, Clot Stiffness of the Quantra in hectopascal (hPa); FCS, Fibrinogen Contribution to Clot Stiffness of the Quantra (hPa); CT, Clot Time in seconds (sec) of the Quantra; CTH, Heparinase Clot Time (sec) of the Quantra; PCS, Platelet Contribution to Clot Stiffness (hPa) calculated by subtracting FCS from CS of the Quantra. EXTEM A10, clot stiffness of the ROTEM after 10 min running time (hPa); FIBTEM A10, fibrinogen contribution to clot stiffness of the ROTEM after 10 min running time (hPa); INTEM CT, Clot time of intrinsic pathway of the ROTEM (sec); HEPTEM CT, Clot time of the intrinsic pathway after neutralization of heparin of the ROTEM (sec); EXTEM A10 – FIBTEM A10, Platelet contribution to clot stiffness of the ROTEM after 10 min running time (hPa); Platelets, platelet count (10^9^/L).

Table 4 shows that the proportion of clot times above the reference ranges is found much more frequently in the ROTEM sigma compared with the Quantra in both sample 1 and sample 2. Expressed in hPa, the Quantra clot stiffness parameters were 3 to 5-fold higher than those of the ROTEM as shown in the scatter plots for clot stiffness parameters with the 3 to 4 times larger unit range abscissa compared to the ordinate (Fig. 3).

**Table 4:** Comparison clotting times of the Quantra and the ROTEM

Parameter	Median [IQR] sample 1	Outside Reference Range x/n	Median [IQR] sample 2	Outside Reference Range x/n	Reference Range
CT	142 [132; 154] sec	3/38	153 [137; 166] sec	11/35	113–164 s
CTH	134 [126; 146] sec	3/38	139 [131;157] sec	14/35	109–150 s
INTEM-CT	172 [161; 183] sec	16/38	232 [232; 305] sec	35/35	138–174 s
HEPTEM-CT	168 [160; 181] sec	15/38	267 [232; 301] sec	34/35	45–173 s

*Abbreviations*: *CT* Coagulation time of the intrinsic coagulation pathway of the Quantra in seconds, *CTH*, Clot time in the presence of heparinase 1 of the Quantra in seconds, *INTEM-CT* Clot time of the ROTEM in seconds (time from start the test until a clot firmness of 2 mm is detected), *HEPTEM-CT* Clot time of INTEM added with heparinase 1 of the ROTEM in seconds

Very Strong correlations were found for EXTEM A10 with CS and for FIBTEM A10 with FCS. INTEM CT and CT in both samples, the platelet count with EXTEM A10 – FIBTEM A10 in sample 1, HEPTEM CT and CTH, and EXTEM A10 – FIBTEM A10 and PCS in sample 2 showed strong correlations. (Table 5, Fig. 3). Moderate correlations were found for HEPTEM CT and CTH in sample 1 and platelet count with EXTEM A10 – FIBTEM A10 and platelet count with PCS in sample 2. Only weak correlations were observed for EXTEM A10 – FIBTEM A10 and PCS and for platelet count and PCS in sample one (Table 5, Fig. 3). There was strong evidence for a difference in the Spearman’s rank-order correlation coefficients for EXTEM A10 – FIBTEM A10 and PCS between sample 1 with r = 0.15 and 2 with r = 0.80 (*p* = 0.0002).

**Table 5:** Spearman’s rank-order correlation between ROTEM and Quantra

Comparison	r (95% CI) sample 1 (*n* = 37)	r (95% CI) sample 2 (*n* = 35)	r diff sample 1 to sample 2 (95% CI)	r diff *p*-value*
EXTEM A10 (hPa) vs Quantra CS (hPa)	0.93 (0.86 to 0.96)	0.94 (0.88 to 097)	− 0.01 (− 0.08 to 0.05)	1.00
FIBTEM A10 (hPa) vs Quantra FCS (hPa)	0.92 (0.85 to 0.96)	0.96 (0.92 to 0.98)	− 0.04 (− 0.11 to 0.01)	0.60
INTEM CT (sec) vs Quantra CT (sec)	0.71 (0.51 to 0.84)	0.75 (0.55 to 0.86)	−0.03 (− 0.28 to 0.20)	1.00
HEPTEM CT (sec) vs Quantra CTH (sec)	0.59 (0.33 to 0.77)	0.77 (0.59 to 0.88)	−0.18 (− 0.47 to 0.08)	0.68
(EXTEM A10 – FIBTEM A10) (hPa) vs Quantra PCS (hPa)	0.15 (−0.18 to 0.45)	0.80 (0.64 to 0.90)	−0.65 (− 0.99 to − 0.33)	0.0002
Platelet count (×1000/μL) vs (EXTEM A10 – FIBTEM A10) (hPa)	0.70 (0.48 to 0.83)	0.69 (0.46 to 0.83)	0.01 (−0.23 to 0.25)	1.00
Platelet count (×1000/μL) vs Quantra PCS (hPa)	0.37 (0.05 to 0.62)	0.69 (0.46 to 0.83)	−0.32 (− 0.65 to − 0.01)	0.28

95% CI, 95% confidence interval, r diff; difference between regression coefficient of sample 1 and 2

For ROTEM parameters EXTEM and FIBTEM, A10 refers to amplitude at 10 min

Quantra parameters: CS, clot stiffness; FCS, fibrinogen contribution to clot stiffness; PCS, platelet contribution to clot stiffness; CT, clot time; CTH, clot time with heparinase

* *P*-values for the comparison of correlations between samples were calculated according to Zou and Silver et al. [16] and adjusted for multiple testing by the Holm-Bonferroni method [17]

**Fig. 4:**
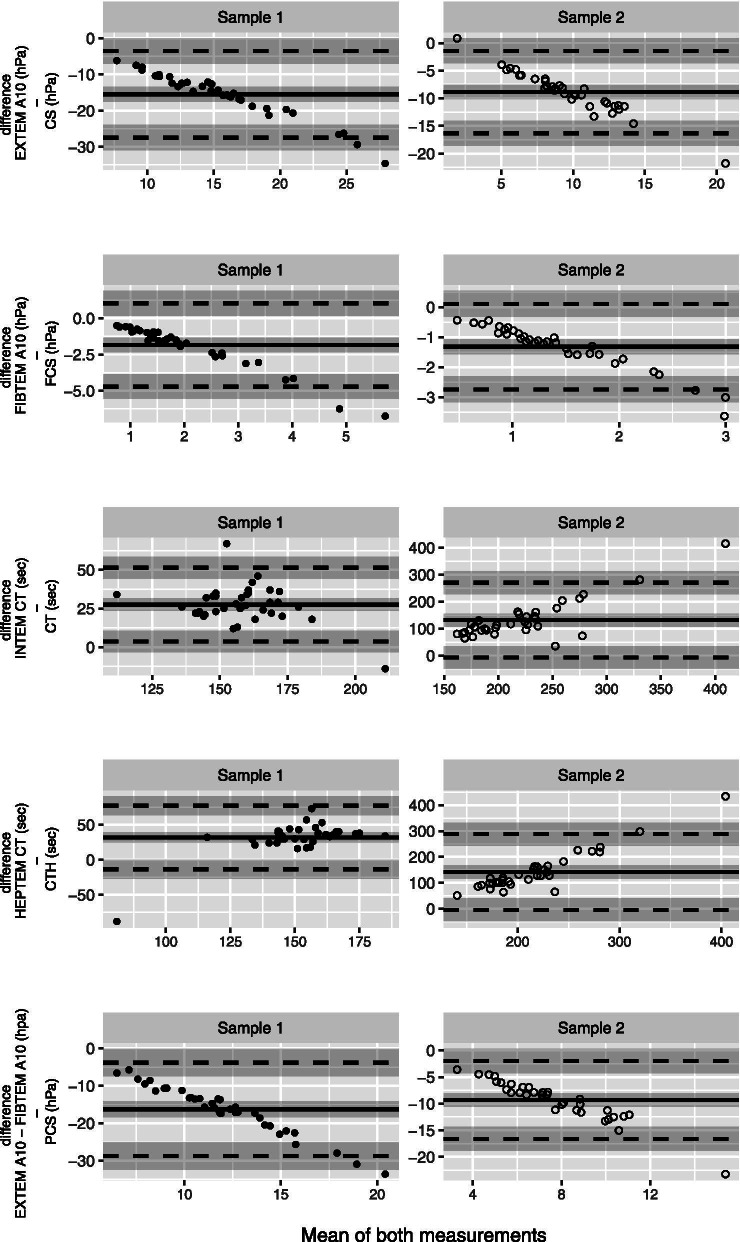
Bland Altman analysis. Plots for comparison of corresponding measurements of the Quantra and ROTEM sigma for sample 1 and 2 (EXTEM A10 and CS; FIBTEM A10 and FCS, INTEM CT and CT, HEPTEM CT and CTH; EXTEM A10-FIBTEM A10 and PCS). Data pairs of sample 1 are presented as solid circles and data pairs of sample 2 are presented as open circles. Y-axis represents the bias (difference of the methods) and X-axis the mean of both measurements. The mean bias (mean difference of the methods) is shown as solid line and the 95% limits of agreement is shown by the dashed lines. *Abbreviations:* CS, Clot Stiffness of the Quantra in hectopascal (hPa); FCS, Fibrinogen Contribution to Clot Stiffness of the Quantra (hPa); CT, Clot Time in seconds (sec) of the Quantra; CTH, Heparinase Clot Time (sec) of the Quantra; PCS, Platelet Contribution to Clot Stiffness (hPa) calculated by subtracting FCS from CS of the Quantra. EXTEM A10, clot stiffness of the ROTEM after 10 min running time (hPa); FIBTEM A10, fibrinogen contribution to clot stiffness of the ROTEM after 10 min running time (hPa); INTEM CT, Clot time of intrinsic pathway of the ROTEM (sec); HEPTEM CT, Clot time of the intrinsic pathway after neutralization of heparin of the ROTEM (sec); EXTEM A10 – FIBTEM A10, Platelet contribution to clot stiffness of the ROTEM after 10 min running time (hPa); Platelets, platelet count (10^9^/L)

For all corresponding measured parameters of the ROTEM and the Quantra, Bland-analysis showed no agreement (Table supplement 2, Fig. 4). The Bland Altman plots (i.e. the relation between the difference (bias) and the mean of both measurements) for EXTEM A10 and CS, FIBTEM A10 and FCS, EXTEM A10 – FIBTEM A10 and PCS showed a nearly linear slope in both samples, indicating that the bias increased with the mean. For INTEM CT and CT and HEPTEM CT and CTH, the mean bias increased 4-fold in the second sample. Spearman’s rank correlation between the various variables shows that only the platelet count measured after arrival in the ICU shows a weak association with the 6 h blood loss with r [CI95] of 0.313 [− 0.022 to 0.585].

## Discussion

This is the first investigation comparing the fully automated viscoelastic point-of-care Quantra and ROTEM sigma analyzers. For both samples tested, after induction of anesthesia and after termination of cardiopulmonary bypass (CPB) and heparin, strong correlations were observed for the clot times and, in particular, for the clot stiffness parameters. These results largely agree with those of the few previous studies that have compared Quantra against the predecessor version, the ROTEM delta. However, Bland Altman analyses of all corresponding parameters showed no agreement between the two methods. This indicates that the results of both devices are not interchangeable.

Huffmyer et al. compared corresponding parameters of the Quantra and the ROTEM delta device in 55 elective cardiac surgery patients [7]. The clot stiffness parameters reported strong correlations (*r* = 0.85 and *r* = 0.84) for all time points, while the correlation of the clot times was only strong for measurements taken before CPB was started. In a comparative study of the same devices in 30 cardiac surgery patients, Baryshnikova et al. found strong correlations (*r* = 0.87–0.96) for the clot stiffness parameters in all samples but only weak to moderate correlations for the clot times [8]. Most recently, strong correlations for corresponding clot times and clot stiffness parameters were reported by Groves et al. in a multicenter study of 277 adult cardiac, major orthopedic or non-surgical patients with coagulation disorders [9]. Additionally, they showed that the correlation of the parameters measuring the platelet contribution to clot stiffness in both devices was only weak for the measurements after induction of anesthesia but strong for those measured after neutralization of the heparin effect. Similar results were reported by Huffmyer et al. who compared the Quantra results with those of the ROTEM delta device [7]. In this investigation, the comparison of the platelet count with the platelet contribution to clot stiffness (PCS) of the Quantra showed, similarly to previous studies, only a weak correlation in the measurements after induction of anesthesia and a moderate correlation after neutralization of the heparin effect. Baryshnikova et at al., indicated an independent - but only weak - association of the PCS of the Quantra with the platelet count and ADP-dependent platelet function as measured by multiple electrode aggregometry, which might explain these findings [8]. In nearly 70% of the patients, the antiplatelet drugs were not discontinued preoperatively on time. The use of the cardiopulmonary bypass during cardiac surgery could wash out a part of the antiplatelet drugs and thus reduce its impact on platelet function.

Correlation coefficients describe the strength and direction of an association between variables, while Bland Altman analysis determine two quantitative measurements’ equivalence or agreement. Even if the correlation of the clot stiffness and clot time parameters in this study was strong, no agreement between the methods was found. Bland Altman analyses of all compared corresponding parameters showed a nearly linear slope in both samples. These findings indicate that results of both devices are not interchangeable, which might be explained by substantial differences in methods used by the two viscoelastic test devices. It could be hypothesized that the oscillation of the pin causes a delay in clot formation by mechanical tearing the resulting clot during the early phase. In contrast, the high-frequency ultrasound impulses only cause the developing clot to move. This hypothesis might be supported by the higher clot stiffness and shorter clot time values of the Quantra compared to the corresponding measures of the ROTEM sigma. In addition, different reagents and different concentrations are used to measure clot time, clot stiffness and fibrinogen contribution to clot stiffness. Finally, the conversion of the unit “mm” into “hPa” to compare the clot stiffness parameters of the ROTEM with those of the Quantra could cause a proportional error, which is reflected in the almost linear slope in the Bland-Altman plots for both samples. Recently, Hochleitner et al. revised the Hartert’s 1962 calculation of the shear modulus of a routine blood clot for thromboelastography and thromboelastometry and reported improved accuracy versus earlier calculation [22]. However, the effect of applying the revised Hartert’s calculation on the clot stiffness parameter of the ROTEM in this investigation is too weak to improve the agreement significantly.

ROTEM’s clot times above the reference range are significantly more frequently observed compared with the Quantra. While clot times of the ROTEM in the measurements after neutralization of the heparin effect were above the normal reference range in nearly 100%, more than 70% of the clot times of the Quantra were within the normal reference range. This finding is in agreement with other studies comparing Quantra CT with ROTEM delta INTEM CT [8]. In the four bleeding patients of our study, the clot times were prolonged in both devices. Nevertheless, prolonged clot times of the ROTEM were also found in 29 not significantly bleeding patients. In contrast, in only 7 not significantly bleeding patients, the clot times of the Quantra were above the normal reference range. However, results outside the predefined reference range do not necessarily trigger hemostatic treatment because established treatment algorithms for using and interpreting the results of thromboelastography and thromboelastometry devices are based on the clinical signs and expert opinion. Additionally, to what extent the clot time measurements of the Quantra could have a higher sensitivity and specificity for predicting the current coagulation situation of the patient has to be clarified by future investigations.

The delivery time of the first and the complete results was considerably shorter for the Quantra compared to the ROTEM, which is explained by the different techniques of the cartridge processing in both devices. While tests are performed successively in each channel of the cartridge in the ROTEM, the test processing in the Quantra cartridge takes place simultaneously. As a result, the Quantra does not provide a number of the clot amplitude after 5, 10, 20 until 60 min running time (A5, A10, A20 until A60). Furthermore, the Quantra cartridge has a shorter sample warming time (3 min vs 8 min of the ROTEM sigma). Additionally, the different reagents and their concentrations should also have considerable influence on the turnaround times of both devices. For the difference between the time to first results of sample 1 and 2, the higher reference range of the clotting times of the ROTEM compared to the Quantra could be another explanation. The heparin:protamine ratio for neutralization of the heparin effect after finishing the CPB might have an additional impact on the turnaround time of the ROTEM device. In this investigation, the heparin was neutralized with protamine in a protamine to heparin ratio of 1:1. It is well known that overdose of protamine has anticoagulant effects, leading to an elongation of either the INTEM-CT and/or HEPTEM-CT. Therefore, it cannot be ruled out that the difference between the time to complete results of the sample 1 and 2 could be caused by a different sensitivity to detect a protamine overload-based coagulopathy. The faster delivery of results by the Quantra suggests faster and more timely decision-making regarding coagulation treatment, provided that the measured values adequately depict the clinical coagulation process.

### Limitations

There are several limitations to this study. First, the small sample size, unfortunately, does not allow any statistically evaluable associations to clinical parameters. Second, in addition to the comparative investigation of measurement parameters of the two devices, no standard coagulation lab tests were carried out at the measurement time points. Third, for comparison of the PCS and the EXTEM A10 – FIBTEM A10 values with platelet count, the preoperative and postoperative platelet count was used. The comparison of platelet counts measured in real-time could have provided more precise results. Finally, this investigation was performed in elective cardiac surgery patients, so the results found in this study cannot be easily transferred to other cohorts of surgical patients.

## Conclusions

The Quantra showed a strong correlation with the ROTEM sigma for determining clot times and clot stiffness, particularly after heparin’s neutralization. However, the Quantra measurements were not directly interchangeable with those of the ROTEM sigma, which implicates that device-specific cut-off values need to be established for users of the Quantra device in a prospective study.

## Supplementary Information

**Additional file 1.**

**Additional file 2.**

## Data Availability

The data used to support the findings of this study are available from the corresponding author upon request.
