# Supplementary material for: Comparison of the resonance sonorheometry based Quantra® system with rotational thromboelastometry ROTEM® sigma in cardiac surgery – a prospective observational study
Source: BMC Anesthesiol. 2021 Oct 28;21:260. doi: 10.1186/s12871-021-01469-5 (PMC8555139; doi:10.1186/s12871-021-01469-5)
Supplement: Supplementary file 1 — Additional file 1. [file 12871_2021_1469_MOESM1_ESM.docx]

**Table supplement 1.** Turnaround time comparison between ROTEM and Quantra.

|  | Sample 1+2 (n=72) | | | Sample 1 (n=37) | | Sample 2 (n=35) | |
| --- | --- | --- | --- | --- | --- | --- | --- |
| Time | ROTEM | Quantra | Estimate, 95 % CI, p-value* | ROTEM | Quantra | ROTEM | Quantra |
| t1, sec | 60 [32;70] | 66 [55;84] | -11.85 from -25.16 to 1.50, 0.08 | 58 [22;68] | 71 [59;84] | 60 [39;73] | 63 [53;81] |
| t2, sec | 658 [635;732] | 412 [396;434] | 293.89 from 252.54 to 335.20,  < 0.0001 | 636 [621;654] | 410 [396;431] | 721 [665;781] | 412 [400;439] |
| t3, sec | 1290 [1264;1345] | 839 [810;897] | 461.32 from 437.95 to 484.70, < 0.0001 | 1264 [1255;1280] | 816 [802;827] | 1343 [1305;1383] | 865 [849;897] |

Data are presented as median [IQR].

Abbreviations: t1, time to cartridge (time needed from blood sampling until insertion of the cartridge); t2, time to first results (time needed until first results are available; t3, time to end results (time needed until complete results are available

* Estimate of the difference in means with 95 % CI, derived by paired t-tests over the joined measurements of sample 1 and sample 2.
