# Supplementary material for: Comparison of the resonance sonorheometry based Quantra® system with rotational thromboelastometry ROTEM® sigma in cardiac surgery – a prospective observational study
Source: BMC Anesthesiol. 2021 Oct 28;21:260. doi: 10.1186/s12871-021-01469-5 (PMC8555139; doi:10.1186/s12871-021-01469-5)
Supplement: Supplementary file 2 — Additional file 2. [file 12871_2021_1469_MOESM2_ESM.docx]

Table supplement 2. Bland Altman Analysis of ROTEM and Quantra comparison.

| Difference | S1 mean | S1 SD | S1 LOA | S2 mean | S2 SD | S2 LOA |
| --- | --- | --- | --- | --- | --- | --- |
| mean EXTEM A10 - mean CS, hPa | -15.5 | 6.1 | -27.7; -3.3 | -8.9 | 3.9 | -16.6; -1.2 |
| mean FIBTEM A10 - mean FCS, hPa | -1.8 | 1.5 | -4.78; 1.1 | -1.3 | 0.7 | -2.8; 0.1 |
| mean INTEM CT - mean CT, sec | 27.5 | 12.2 | 3.3; 51.9 | 131.5 | 71.4 | -11.3; 274.3 |
| mean HEPTEM CT – mean CTH, sec | 31.9 | 23.1 | -14.3; 78.1 | 141.8 | 75.5 | -9.2; 292.8 |
| Mean (EXTEM A10-FIBTEM A10) – mean PCS, hPa | -16.3 | 6.4 | -29.0; -3.6 | -12.0 | 4.8 | -21.5; -2.4 |

Abbreviations: EXTEM A10, FIBTEM A10, INTEM A10, HEPTEM A10 are parameters of the ROTEM, CS, FCS, CT, CTH and PCS are parameters of the Quantra; S1 mean, mean bias of sample 1 (n=37); S1 SD, standard deviation of sample 1; S1 LOA, Limits of agreement of sample 1; S2 mean, mean bias of sample 2 (n=35), S2 SD, standard deviation of sample 2; S2 LOA, limits of agreement of sample 2;

For ROTEM parameters EXTEM and FIBTEM, A10 refers to amplitude at 10 min.

Quantra parameters: CS, clot stiffness; FCS, fibrinogen contribution to clot stiffness; PCS, platelet contribution to clot stiffness; CT, clot time; CTH, clot time with heparinase.
